# Long-Term Symptoms of COVID-19: One-Year Follow-Up Study

**DOI:** 10.7759/cureus.25937

**Published:** 2022-06-14

**Authors:** FNU Shivani, Naina Kumari, Pinky Bai, FNU Rakesh, Muhammad Haseeb, Sunny Kumar, Amna Jamil, Maheen Zaidi, Faizan Shaukat, Amber Rizwan

**Affiliations:** 1 Internal Medicine, Peoples University of Medical and Health Sciences, Nawabshah, PAK; 2 Internal Medicine, Dow University of Health Sciences, Karachi, PAK; 3 Internal Medicine, Jinnah Sindh Medical University, Karachi, PAK; 4 Internal Medicine, Bahria International Hospital, Lahore, PAK; 5 Internal Medicine, Liaquat University of Medical and Health Sciences, Jamshoro, PAK; 6 Internal Medicine, Jinnah Postgraduate Medical Center, Karachi, PAK; 7 Family Medicine, Jinnah Postgraduate Medical Center, Karachi, PAK

**Keywords:** multisystem, pneumonia, sars-cov-2 (severe acute respiratory syndrome coronavirus -2), long-covid-19, covid-19

## Abstract

Introduction: Persistent and prolonged symptoms, termed as long COVID (coronavirus disease), have been reported in several patients who recovered from the acute phase at different intervals. However, there has been largely unclear data regarding the full range of long-term sequelae of coronavirus disease 2019 (COVID-19) patients. This study aims to evaluate the prevalence of long COVID syndrome.

Methods: A long-term research was conducted in the COVID-19 unit of a tertiary care hospital in Pakistan from July 2020 to December 2021 in which 2,000 patients who had recovered from COVID-19 and had been discharged were included in the study. Symptoms were noted at the time of discharge and at follow-up after 12 months. Data were analyzed using Statistical Package for the Social Sciences (SPSS) v. 22.0 (IBM Corporation, Armonk, New York, United States).

Results: The mean age of the participants was 43 ± 10 years, 801 (53.8%) males and 688 (46.2%) females. At the time of discharge, the most common symptom was fatigue (26.93%), followed by dyspnea (20.34%) and muscle pain (8.86%). The most common symptom on follow-up was fatigue (6.78%).

Conclusion: We strongly emphasize discussing and exploring further knowledge on the post-infection syndrome, with an aim to bring healthcare professionals’ attention to the importance of handling COVID patients, their counseling, warning for alarming signs, and a long-term follow-up with necessary investigations and treatment.

## Introduction

The coronavirus disease 2019 (COVID-19) pandemic started in late 2019, causing a huge global disease burden [1]. Belonging to the family of coronaviridae, severe acute respiratory distress syndrome coronavirus 2 (SARS-CoV-2) has been identified as the culprit behind this pandemic, causing significant morbidity and mortality [2]. The acute presentation of COVID-19 ranges from asymptomatic infection to mild flu-like symptoms from which most patients recover successfully; however, it can cause pneumonia leading to sepsis with life-threatening outcomes, particularly in the elderly population [3]. 

In addition, persistent and prolonged symptoms, termed as long COVID, have been reported in several patients who recovered from the acute phase at different intervals. According to the National Institute for Health and Care Excellence (NICE), the definition of long COVID is the presence of signs and symptoms that develop during or after an infection consistent with COVID-19, continuing for more than 12 weeks, and are not explained by an alternate diagnosis [2]. These include weakness, myalgias, sleep disturbances, shortness of breath, loss of taste and smell, headaches, physical and psychological disturbances, and cognitive impairment [4,5]. Moreover, health-related quality of life (HRQoL) has been reported to decrease in 51-62% of COVID-19 survivors after three to six months [6]. Raised titers of antibodies against SARS, Middle East respiratory syndrome coronavirus (MERS-CoV), and avian influenza A virus (H7N9) have been observed to be present for 12 months [7]. However, there has been largely unclear data regarding the full range of long-term sequelae of COVID-19 patients.

The symptoms may decrease in frequency over months after recovering from the acute phase of COVID-19 but due to the alarmingly increased number of COVID-19 patients, its long-term outcomes are now an increasing concern and hold huge importance in managing and preventing poor outcomes, hence affecting the quality of life and survival.

## Materials and methods

From July 2020 to December 2021, long-term research was conducted in the COVID-19 unit of a tertiary care hospital in Pakistan. After receiving informed consent, 2000 patients who had recovered from COVID-19 and had been discharged were included in the study. Consecutive convenient non-probability sampling was employed as the sampling approach. The ethical review board gave its permission before the subjects were enrolled. Their symptoms were recorded in a self-structured questionnaire at the time of discharge. Participants were told that they will be approached again after a year to inquire about their symptoms.

At follow-up after 12 months, participants were called on the given phone number and inquired about their symptoms. For those who did not receive the call, a minimum of three times were tried to reach out to them. A total of 511 participants were lost to follow-up. Only participants who completed follow-up were included in the final analysis.

Data were analyzed using Statistical Package for the Social Sciences (SPSS) v. 22.0 (IBM Corporation, Armonk, New York, United States). Frequency and percentage were calculated for categorical data. Mean and standard deviation were calculated for numerical data. A p-value of less than 0.05 was considered statistically significant.

## Results

A total of 1489 participants completed the study. The mean age of participants was 43 ± 10 years and the mean length of hospital stay was 4.1 ± 1.1 days. The final analysis included 801 (53.8%) males and 688 (46.2%) females. The inflammatory markers at discharge are mentioned in Table 1.

**Table 1:** Characteristics at the time of discharge CRP: c-reactive protein; ESR: erythrocyte sedimentation rate; LDH: lactate dehydrogenase; mg/L: milligrams per liter; mm/h: millimeters per hour; SD: standard deviation.

Characteristics at the time of discharge	Frequency (%)/Mean ± SD (n= 1498)
Age (in years)	43 ± 10
Gender	
Male	801 (53.8%)
Female	688 (46.2%)
Length of hospital stay (days)	4.1 ± 1.1
ESR at discharge (mm/h)	11.2 ± 3.9
CRP at discharge (mg/L)	11.1 ± 2.7
LDH at discharge (IU)	317.2 ± 89.2

At the time of discharge, the most common symptom was fatigue (26.93%), followed by dyspnea (20.34%), muscle pain (8.86%), and headache (7.52%). Other symptoms at the time of discharge are mentioned in Table 2.

**Table 2:** Frequency of symptoms at the time of discharge

Symptoms	Frequency (n= 1489)
Fatigue	401 (26.93%)
Dyspnea	303 (20.34%)
Muscle pain	132 (8.86%)
Headache	112 (7.52%)
Insomnia	110 (7.38%)
Anosmia	29 (1.94%)
Ageusia	28 (1.88%)
Altered vision	20 (1.34%)
Dizziness	19 (1.27%)
Confusion	18 (1.20%)

The most common symptom on follow-up was fatigue (6.78%), followed by dyspnea (4.56%). Among the neurological symptoms, headache was most common (1.34%), followed by insomnia (1.14%). Other symptoms at the time of discharge are mentioned in Table 3.

**Table 3:** Frequency of symptoms at follow-up

Symptoms	Frequency (n= 1489)
Fatigue	101 (6.78%)
Dyspnea	68 (4.56%)
Muscle pain	27 (1.81%)
Headache	20 (1.34%)
Insomnia	17 (1.14%)
Anosmia	12 (0.80%)
Ageusia	08 (0.53%)
Dizziness	02 (0.13%)
Confusion	02 (0.13%)
Altered vision	01 (0.06%)

## Discussion

The rapidly rising number of reported cases with persistent COVID-19 symptoms after the remission of original infection brings about huge attention to a term called “Post-COVID Syndrome (PCS)”. A cohort study observed that severely ill patients during hospital stay had an increased risk of lung diffusion impairment, radiographic abnormality, and muscle or limb pain than those with less severity of symptoms [8]. PCS can range from mild symptoms like insomnia, general myalgia, fatigue, and dry skin to severe neurological, cardiopulmonary, and musculoskeletal disorders, including stroke, heart failure, myalgic encephalomyelitis, and chronic fatigue syndrome [9,10].

Our study reported the rates of acute and long-term symptoms consistent with COVID-19 after a thorough analysis during the discharge of previously confirmed COVID patients and then after a year of follow-up. According to our study, fatigue (26.93% vs. 6.78%), dyspnea (20.34% vs. 4.56%), and muscle pain (8.86% vs. 1.81%) were noticed to be among the most common symptoms present on the discharge day as well as on one-year follow-up in COVID-19 recovered patients. Headache has also been found to be a frequent complaint among sufferers that goes along for a more extended period of time, i.e. 7.52% on discharge and 1.34% after a year. A study reported dyspnea and fatigue to be the most common acute symptoms that are often associated with joint or chest pain [11]. In addition to these general symptoms, the involvement of major organs like the heart, lung, and brain, causing myocardial inflammation, myocarditis, cardiac arrhythmias, interstitial thickening and fibrosis, headache, vertigo, encephalitis, seizures, major mood swings, and brain fog [12,13], have been reported with cases of end-organ damage [14].

Another online survey conducted by the British Medical Association among healthcare professionals reported that a third of doctors claimed that they have seen and treated patients with symptoms they believe to be the long-term effect of COVID-19, including muscle fatigue, anosmia, and concentration difficulties [15]. However, arthralgia and diarrhea persisted in more than 40% of PCS patients [16]. In a meta-analysis, 15 studies stated fatigue as a common symptom of long COVID, with a pooled analysis indicating fatigue and dyspnea to be reported by 38% and 32% of the people, respectively [17]. Up to 59% of patients showed persistent chest computed tomography abnormalities at a mean of 3.8 months follow-up [17].

The involvement of neuropsychiatric sequelae by viral pathogens has always been a highlight when discussing long-term complications and persisting symptoms in COVID-19 recovered patients [13]. Ample shreds of evidence with neurological deficits and cognitive decline have been reported in a large population after overcoming the primary COVID infection that can affect the overall well-being and can seriously threaten cognitive health and mental function [18]. Most of the previous literature, to date, has defined headache, vertigo, and chemosensory dysfunction like anosmia and ageusia to be the most common long-term neurological symptoms to persist after COVID-19 infection [13]. This strongly supported the results of our studies that show the headache to persist in about 1.34%, anosmia in 0.8%, and ageusia in 0.53% on post-COVID follow-up.

Besides clinical symptoms, studies have also brought some light to emotional and behavioral concerns and general distress that comes with the social distancing, isolation, and COVID-related stigma that puts the patient at great risk of depression, chronic fatigue syndrome, and other psychological disturbances, including suicidal behavior [19,20]. 

According to a study, 87% of the COVID patients recovered, and persistence of at least one to two symptoms was observed in 32% of the patients while the rest demonstrated three to four symptoms after two months of recovery [21]. Commonly observed symptoms were fatigue (53%), low HRQoL (44.1%), dyspnea (43%), and arthralgia (27%) [21]. Other long-term serious consequences are dysregulated sleep-wake cycle, cognitive impairment, profound unremitting anergia, and chronic relapses of exhaustion together termed “post-exertional malaise” [22,23]. Any such persisting symptom after recovery from SARS-CoV-2 should be seriously considered and treated as they may reflect severe post-COVID syndrome [24]. Of the severely ill patients, 59.4% showed symptoms that persisted for more than 30 days, whereas it was 14.3% in patients with mild to asymptomatic infection [25]. This strongly suggested the correlation between the severity of the infection and the development of long-term complications.

Our study was underpowered to identify the factors and other underlying comorbidities that can have a direct correlation with the development of long-term complications in COVID-19 recovered patients. Though, we do identify the most common symptoms present on the discharge day, and their persistence rate after a year. The symptoms were self-assessed rather than focused on measured via any validated scale. This stratification elucidates “short term” and “long term” COVID-19 symptoms. Despite our strengthful population-level survey, our study limits our potentiality to anticipate the association of long-term complications with the severity of the infection. 

## Conclusions

As our experience with this virus continues to grow, our understanding of both short-term and long-term complications of infection with SARS-CoV-2 is growing continuously. By thoroughly assessing the patient's condition, symptoms present on the discharge day of COVID recovered patients and then following them after a year gives us the information about the persisting symptoms. Knowing all the previous data and literature, we strongly emphasize discussing and exploring further knowledge on the post-infection syndrome, with an aim to bring healthcare professionals’ attention to the importance of handling COVID patients, their counseling, warning for alarming signs, and a long-term follow-up with necessary investigations and treatment. In order to achieve that, healthcare planning, resource allocation, and the role of a multidisciplinary team of professionals are vital; post-COVID-19 rehabilitation programs and centers should be set up and easily accessible in different areas where COVID was widely spread. In this manner, patients will be closely monitored and provided with necessary measures to combat the after-effects.
